# Association between oxidative balance score and all-cause, CVD and respiratory-related mortality in the US older adults of asthma patients with diabetes

**DOI:** 10.3389/fnut.2024.1519570

**Published:** 2025-01-15

**Authors:** Chang Liu, Dan Liang, Guoan Xiang, Xuanbo Zhao, Kun Xiao, Lixin Xie

**Affiliations:** ^1^School of Medicine, Nankai University, Tianjin, China; ^2^Department of Endocrine, People's Hospital of Chongqing Liang Jiang New Area, Chongqing, China; ^3^West China Medical College of Sichuan University, Chengdu, China; ^4^College of Pulmonary and Critical Care Medicine, Chinese People's Liberation Army (PLA) General Hospital, Beijing, China; ^5^Clinical Medicine College of Henan University of Traditional Chinese Medicine, Zhengzhou, China

**Keywords:** diabetes, NHANES, all-cause mortality, CVD mortality, OBS

## Abstract

**Background:**

This study aims to investigate the correlation between oxidative balance score (OBS) and all-cause, cardiovascular disease (CVD) and respiratory-related mortality within a cohort that includes older asthma patients with diabetes.

**Methods:**

Data from the National Health and Nutrition Examination Survey (NHANES) spanning from 2001 to 2018, which included 611 participants, were analyzed. Mortality outcomes were determined by linking the data to National Death Index (NDI) records through December 31, 2019. Cox regression modeling was employed to examine the relationship between OBS and all-cause, CVD and respiratory-related mortality. Restricted cubic splines (RCS), subgroup analyses and interaction tests were also conducted in this study.

**Results:**

Over a median follow-up of 78.96 months, there were 216 all-cause deaths and 57 CVD-related deaths. A significant negative association was found between the OBS and all-cause and CVD mortality. We did not observe OBS could reduce respiratory-related mortality in older asthma patients with diabetes. RCS analysis indicated a linear and inverse association between the OBS and all-cause and CVD mortality. Subgroup analyses and interaction tests indicated the negative association between OBS and CVD mortality was significantly influenced by alcohol consumption.

**Conclusion:**

In this sample, higher OBS was associated with lower all-cause and CVD mortality risks. These findings stressed the importance of infection status in assessing oxidative balance’s impact on health.

## Introduction

Asthma is a prevalent global chronic non-communicable disease marked by bronchospasm and airway inflammation, affecting approximately 334 million people worldwide (1). It accounts for 1.1% of global disability-adjusted life years (DALYs) (2). Common symptoms include dyspnea, chest tightness, and wheezing (2). Asthma also elevates the risk of cardiovascular disease and mortality, with its prevalence increasing after age 50, peaking at 6.8–12% (3–6). This trend suggests a growing socioeconomic and healthcare burden. Diabetes mellitus, characterized by elevated blood glucose levels, affected 451 million people globally in 2017, with projections estimating 693 million adults with diabetes by 2045 (7). People with diabetes are at higher risk for asthma and other chronic respiratory diseases, including chronic obstructive pulmonary disease (COPD), pulmonary fibrosis, and pneumonia, and this increased risk may be a result of decreased lung function in people with diabetes (8).

Oxidative stress is caused by an imbalance between the cell’s antioxidant scavenging system and the reactive oxygen species (ROS) production process (9). Immune cells involved in the inflammatory response in asthma, such as eosinophils and neutrophils, can produce excessive ROS, contributing to oxidative stress and exacerbating asthma symptoms (10). The oxidative balance score (OBS) is a measure that assesses an individual’s antioxidant status by objectively quantifying the antioxidant and pro-oxidant components of dietary and lifestyle factors (11, 12). Findings suggest a strong association between OBS and reduced all-cause mortality, CVD-related mortality, and cancer-related mortality in populations adhering to the Mediterranean diet (13). One study noted that a composite index (inflammatory burden index) combining multiple inflammatory markers such as C-reactive protein (CRP), neutrophil count and lymphocyte count was independently associated with an increased risk of all-cause as well as respiratory disease mortality in patients with asthma and COPD (14, 15). To improve disease management and health outcomes, it is crucial to understand the impact of OBS on mortality in older asthma patients with diabetes. Therefore, this study aimed to investigate the association between OBS and all-cause, CVD mortality and respiratory-related mortality in older asthma patients with diabetes.

## Materials and methods

The data for this investigation were sourced from the National Health and Nutrition Examination Survey (NHANES) public database, which is managed by the Centers for Disease Control and Prevention (CDC) in the USA. The NHANES database collects vital health statistics for the nation, utilizing a refined and intricate methodology to periodically select a representative sample of the U.S. population. Its primary objective is to assess the health and nutritional status of individuals in the United States. To ensure ethical compliance, the survey has received approval from The National Center for Health Statistics (NCHS) Research Ethics Review Board (ERB; Approval number: Protocol #98-12/Protocol #2005-06/Continuation of Protocol #2005-06/Protocol #2011-17/Continuation of Protocol #2011-17/Protocol #2018–01), and all participants provided written informed consent prior to their inclusion in the study. NHANES encompasses a comprehensive range of data, including demographics, dietary habits, medical examination results, laboratory findings, and questionnaire responses. The data are released in 2-year cycles. The datasets used in this study are publicly accessible on the CDC website.

### Study population

**Figure 1:**
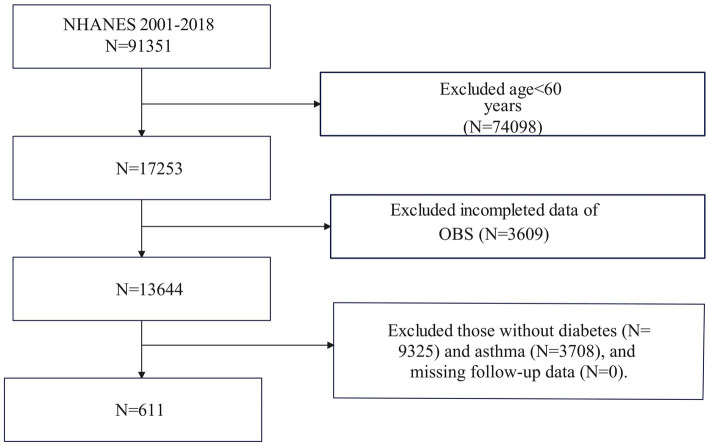
Flowchart of the sample selection from National Health and Nutrition Examination Survey (NHANES) 2001–2018.

Figure 1 illustrates that the NHANES study included 611 participants from 2001 to 2018. Initially, 91,351 participants were enrolled in the study. After excluding individuals under the age of 60 (*N* = 74,098), those with missing data on OBS (*N* = 3,609), and those without diabetes (*N* = 9,325) or asthma (*N* = 3,708), as well as those without follow-up data (*N* = 0), the final analysis comprised 611 eligible participants. Asthma diagnosis in this study was based on responses to two specific questions from the Medical Condition questionnaire: (1) “Have you ever been told you have asthma?” and (2) “Do you still have asthma?” Participants who answered “YES” to both questions were classified as asthma patients; otherwise, they were considered non-asthma patients.

### Assessment of OBS

The OBS was calculated using a comprehensive approach that integrates both dietary and lifestyle factors. The computation involved evaluating 16 nutrients and 4 lifestyle factors that have a known connection with oxidative stress, specifically focusing on 5 pro-oxidants and 15 antioxidants. These components were categorized into four distinct groups to facilitate analysis: (1) Dietary Antioxidants: Fiber, β-carotene, riboflavin, niacin, vitamin B6, total folate, vitamin B12, vitamin C, vitamin E, calcium, magnesium, zinc, copper, and selenium. (2) Dietary Pro-Oxidants: total fat and iron. (3) Lifestyle Antioxidants: physical activity. (4) Lifestyle Pro-Oxidants: alcohol, smoking, and body mass index (BMI). Alcohol consumption was classified into three categories: heavy drinkers (≥15 g/day for women and ≥30 g/day for men), non-heavy drinkers (0–15 g/day for women and 0–30 g/day for men), and non-drinkers, with scores of 0, 1, and 2 points, respectively. Subsequently, the other components were stratified by sex and divided into tertiles. Antioxidants were assigned points on a scale from 0 to 2, with tertile groups 1 to 3 receiving progressively higher scores, indicating increased antioxidant levels. In contrast, pro-oxidants were assigned points in reverse order: the highest tertile received 0 points, and the lowest tertile received 2 points, reflecting higher pro-oxidant levels (12, 16–18). The overall OBS was calculated by summing the points assigned to each component, yielding a total score ranging from 3 to 37. OBS was used in epidemiological studies to consider dietary and non-dietary lifestyle exposures, where pro-oxidants induce oxidative stress by impeding the defences of the antioxidant system (19, 20). And OBS was used as an indicator to assess the overall load of oxidative stress, where an elevated OBS score indicates increased antioxidant exposure. This provides a systematic approach to assess the interaction of different dietary elements and lifestyle choices on oxidative and antioxidant processes in the body (9). This methodical approach allows for a nuanced understanding of individual exposure to both antioxidant and pro-oxidant factors, thereby facilitating personalized health assessments and interventions aimed at reducing oxidative stress.

### Assessment of mortality

To determine the mortality status and causes of death for participants, their records were matched with the National Death Index (NDI) files, ensuring a repetition rate below 10% up to December 31, 2019.^1^ Disease-specific deaths were classified using the International Statistical Classification of Diseases, 10th Revision (ICD-10). Cardiovascular deaths were identified using ICD-10 codes I00–I09, I11, I13, and I20–I51, encompassing conditions such as rheumatic heart disease, hypertensive heart disease, ischemic heart disease, acute myocardial infarction, pericardial disease, acute myocarditis, and heart failure. Respiratory-related mortality was also ascertained by the NCHS through a probabilistic match between NHANES participants and NDI death certificate records encompassing chronic lower respiratory diseases (J40–J47), influenza and pneumonia (J10–J18).

### Assessment of covariates

Data on various demographic and health-related factors were collected through NHANES household interviews. The dataset encompassed age, gender (female/male), race/ethnicity (Mexican American, Non-Hispanic Black, Non-Hispanic White, Others), education levels (less than 9th grade, 9-11th grade, high school graduate, college graduate or above), family income-to-poverty ratio (PIR; <1, 1–4, >4), alcohol use (yes/no), smoking status (never/former/current), and disease status (diabetes/hypertension). Diabetes (DM) was identified by either treatment or a medical diagnosis of hyperglycemia, with criteria including hemoglobin A1c ≥ 6.5%, fasting blood glucose ≥126 mg/dl, or 2-h blood glucose ≥200 mg/dl (21). Hypertension was defined by the use of antihypertensive medications, a medical diagnosis of hypertension, or three consecutive measurements of systolic blood pressure ≥ 140 mmHg or diastolic blood pressure ≥ 90 mmHg (22). BMI was calculated as weight (kg) divided by height squared (m^2^), categorizing participants as normal weight (<25 kg/m^2^), overweight (25–29.9 kg/m^2^), or obese (≥30 kg/m^2^). Laboratory measurements included total cholesterol (TC), triglycerides (TG), fasting glucose, high-density lipoprotein cholesterol (HDL-C), low-density lipoprotein cholesterol (LDL-C), and glycosylated hemoglobin (HbA1c).

### Statistical analysis

Statistical analyses adhered to CDC guidelines, accounting for the complex probability sample design and deliberate oversampling in NHANES to ensure representativeness. Sample weights were applied to integrate data from multiple survey cycles. Participants were categorized into two groups based on survival status (Survival/Death). Continuous variables are presented as means ± standard deviations, while categorical variables are shown as percentages with 95% confidence intervals (CIs). Weighted one-way ANOVA was utilized for continuous variables, and the weighted chi-square test assessed categorical differences during descriptive analyses. Multivariate Cox proportional hazards regression models estimated hazard ratios (HRs) and 95% CIs for the relationship between the OBS and the risk of all-cause and CVD mortality. Three models were constructed, which were as follows: Model 1 was unadjusted; Model 2 adjusted for age, gender, and race; Model 3 adjusted for age, gender, race, education, PIR, BMI, hypertension, total cholesterol, alcohol use, and smoking status (23). Restricted cubic spline (RCS) analysis examined the dose–response relationship between the OBS and all-cause and CVD mortality. In cases of nonlinearity, the threshold value was determined by evaluating all potential values and selecting the one with the highest likelihood. Subgroup analyses of the association between OBS and all-cause and CVD mortality were conducted, stratified by gender (male/female), BMI (normal weight/overweight/obesity), hypertension (yes/no), smoking status (never/former/current), and alcohol use (yes/no), with these factors also considered as potential effect modifiers. Statistical significance was set at two-tailed *p* < 0.05. All analyses were performed using R version 4.3.2 (http://www.R-project.org, The R Foundation).

## Results

### Baseline characteristics of study participants

The study involved 611 older asthma patients with diabetes, and their characteristics were analyzed based on follow-up outcomes (Table 1). The average age of participants was 68.90 ± 0.34 years, with the majority being Non-Hispanic White, and with 58.54% being female. The mean follow-up period was 78.96 months. The number of survivors was 395, and the number of deaths was 216. Statistically significant differences were observed in the distributions of age, total cholesterol, HDL-C, OBS, educational levels, PIR, smoking, and drinking status among the follow-up outcome groups. Compared to the survival group, patients in the death group were older, had higher cholesterol levels, lower OBS, lower levels of education, poorer levels of household income, were more likely to be current smokers and former smokers, and were more likely to be alcohol drinkers. However, no significant differences were found in gender, BMI, triglyceride, LDL-C, fasting glucose, HbA1c, and hypertension (all *p* > 0.05).

**Table 1:** Weighted baseline characteristics of the study population.

OBS	All participants	Survival	Death	*p* value
Age (year)	68.90 (0.34)	67.30 (0.38)	71.30 (0.62)	**<0.0001**
Fastglucose (mg/dl)	141.10 (2.69)	140.64 (3.15)	142.05 (5.34)	0.82
HbA1c	6.84 (0.06)	6.75 (0.07)	6.99 (0.11)	0.07
Total cholesterol (mg/dl)	186.31 (3.09)	185.21 (3.66)	188.40 (5.46)	**0.02**
HDL-C (mg/dl)	50.62 (1.84)	49.48 (1.00)	52.80 (4.84)	**<0.0001**
LDL-C (mg/dl)	103.83 (2.59)	104.32 (3.02)	102.80 (4.80)	0.79
Triglyceride (mg/dl)	157.74 (7.12)	159.05 (9.02)	155.12 (14.87)	0.83
BMI (Kg/m^2)	32.93 (0.42)	33.11 (0.54)	29.42 (0.17)	0.56
OBS	18.32 (0.36)	19.03 (0.43)	17.02 (0.56)	**0.004**
Gender, % (SE)				0.16
Female	58.54 (50.10, 66.98)	61.53 (54.50, 68.29)	53.37 (44.28, 62.45)	
Male	41.46 (33.51, 49.41)	38.65 (31.71, 45.60)	46.63 (37.55, 55.72)	
Races, % (SE)				0.09
Mexican American	4.41 (2.85, 5.97)	5.58 (3.16, 8.01)	2.25 (0.96, 3.54)	
Non-Hispanic Black	14.56 (12.00, 17.12)	14.79 (11.07, 18.51)	14.13 (9.75, 18.52)	
Non-Hispanic White	69.81 (57.79,81.83)	66.63 (60.14, 73.13)	75.67 (68.54, 82.80)	
Others	11.22 (8.19, 14.25)	13.00 (9.27, 16.72)	7.94 (2.32, 13.56)	
Educational levels, % (SE)				**0.04**
Less than 9th grade	9.90 (7.29, 12.52)	8.33 (5.43, 11.24)	12.79 (8.24, 17.35)	
9-11th grade	14.98 (11.41, 18.55)	14.02 (10.41, 17.63)	16.74 (10.96, 22.52)	
High school graduate	25.16 (20.28, 30.04)	22.88 (17.62, 28.14)	29.36 (21.98, 36.74)	
College graduate or above	49.96 (40.95, 58.97)	54.76 (47.96, 61.56)	41.11 (32.50,49.71)	
PIR,% (SE)				**<0.0001**
<1	14.49 (11.49, 17.50)	14.69 (10.53, 18.85)	17.61 (11.05, 24.16)	
1–4	56.05 (47.53, 64.58)	57.16 (49.61, 64.72)	67.43 (58.55, 76.31)	
>4	21.58 (15.13, 28.03)	28.15 (21.13, 35.16)	14.96 (6.42, 23.50)	
BMI, % (SE)				0.68
Normal weight	8.71 (4.92, 12.50)	9.27 (4.04, 14.50)	8.34 (4.16, 12.52)	
Overweight	27.12 (21.48, 32.76)	26.25 (20.30, 32.20)	32.89 (30.41, 35.36)	
Obesity	61.54 (52.56, 70.52)	64.48 (57.49,71.47)	60.79 (52.09, 69.50)	
Smoke, % (SE)				**<0.0001**
Never	41.08 (37.99, 55.33)	47.98 (40.07,55.89)	28.36 (21.53, 35.19)	
Former	46.66 (37.99, 55.33)	42.86 (35.03, 50.68)	53.67 (45.47, 61.87)	
Now	12.26 (8.07, 16.45)	9.16 (5.84, 12.48)	17.97 (9.73, 26.22)	
Alcohol use, % (SE)	62.00 (52.69, 71.31)	66.97 (60.61, 73.34)	52.84 (43.00, 62.67)	**0.02**
Hypertension, % (SE)	84.23 (73.58, 94.88)	84.98 (80.41,89.55)	82.85 (76.97,88.73)	0.57

LDL-C, low-density lipoprotein cholesterol; HDL-C, high-density lipoprotein cholesterol; BMI, body mass index; OBS, oxidative balance score; PIR, family income-poverty ratio. The bold values indicated that the results are statistically significant.

### Association of OBS with all-cause mortality and CVD mortality

Table 2 illustrates the relationship between OBS quartiles and both all-cause and CVD mortality in the study population. Over a median follow-up of 78.96 months, there were 216 all-cause deaths, 57 CVD-related deaths. Three Cox regression models were employed to assess the independent association of the OBS with these mortality risks. In Model 1, a significant negative association was found between the OBS and all-cause mortality (HR:0.96, 95% CI: 0.94–0.98). This negative association persisted in the minimally adjusted Model 2 (HR: 0.96, 95% CI: 0.93–0.98). The association between the OBS and all-cause mortality still remained stable (HR: 0.98, 95% CI: 0.95–0.99), indicating that a 2% decrease in the probability of all-cause death of each increased unit of the OBS. When treating OBS as quartiles, the risk of all-cause mortality was notably lower in the highest OBS quartile compared to the lowest quartile (Model 1: HR: 0.37, 95% CI: 0.23–0.60; Model 2: HR: 0.35, 95% CI: 0.22–0.56). In the fully adjusted Model 3, the HRs (95% CI) for all-cause mortality were 0.54 (0.33–0.88) for quartile 2, 0.77 (0.49–1.21) for quartile 3, and 0.51 (0.29–0.88) for quartile 4, indicating that participants in the highest OBS quartile had a significantly 49% decreased risk of all-cause mortality compared with those in the lowest quartile.

**Table 2:** HRs (95%CI) for mortality according to the OBS.

All-cause mortality		HR (95%CI) *p* value	
	Model 1	Model 2	Model 3
OBS (continuous)	0.96 (0.94, 0.98) **0.001**	0.96 (0.93, 0.98) **0.002**	0.98 (0.95, 0.99) **0.001**
OBS (quartiles)			
Quartile 1	Reference	Reference	Reference
Quartile 2	0.45 (0.28, 0.72) **<0.001**	0.39 (0.26, 0.59) **<0.0001**	0.54 (0.33, 0.88) **0.01**
Quartile 3	0.55 (0.37, 0.82) **0.003**	0.55 (0.37, 0.83) **0.005**	0.77 (0.49, 1.21) 0.26
Quartile 4	0.37(0.23, 0.60) **<0.0001**	0.35 (0.22, 0.56) **<0.0001**	0.51 (0.29, 0.88) **0.02**
CVD mortality		HR (95%CI) *p* value	
OBS (continuous)	0.95 (0.91, 0.99) **0.02**	0.93 (0.89, 0.98) **0.003**	0.95 (0.90, 0.99) **0.04**
OBS (quartiles)			
Quartile 1	Reference	Reference	Reference
Quartile 2	0.58 (0.26, 1.30) 0.19	0.40 (0.19, 0.84) **0.02**	0.47 (0.18, 1.22) 0.12
Quartile 3	0.43 (0.20, 0.96) **0.04**	0.35 (0.16, 0.76) **0.01**	0.49 (0.19, 1.26) 0.14
Quartile 4	0.21 (0.08, 0.54) **0.001**	0.16 (0.07, 0.41) **<0.0001**	0.19 (0.06, 0.58) **0.003**

HR, hazard ratio; 95%CI, 95% Confidence Interval. Model 1 was unadjusted; Model 2 adjusted for age, gender, and race; Model 3 adjusted for age, gender, race, education, PIR, BMI, hypertension, fasting total cholesterol, alcohol use, and smoking status. The bold values indicated that the results are statistically significant.

For CVD mortality, a negative association with the OBS was observed in both Model 1 (HR: 0.95, 95% CI: 0.91–0.99) and Model 2 (HR: 0.93, 95% CI: 0.89–0.98). Even after adjusting for potential confounders in Model 3, this association was still significant (HR: 0.95, 95% CI: 0.90–0.99). Even after treating the OBS as quartiles, there was still a statistically significant association. A significant 81% lower risk of all-cause mortality was experienced by subjects in the highest OBS quartiles compared to those in the lowest OBS quartile (HR: 0.19, 95% CI: 0.06–0.58).

### Association of lifestyle OBS and dietary OBS with all-cause and CVD mortality

Supplementary Table 1 displays the correlation between lifestyle OBS and the risk of all-cause and CVD mortality. After adjusting for Model 3, there was a significant negative association between lifestyle OBS and the risk of all-cause mortality (HR: 0.75, 95%CI: 0.62–0.91). However, no significant inverse relationship was observed between lifestyle OBS and CVD mortality (HR: 0.88, 95%CI: 0.61–1.28). When lifestyle OBS was categorized into quartiles, similar negative associations were observed compared to continuous lifestyle OBS. The adjusted HR for Quartile 4 in model 3 was 0.47 (95%CI: 0.23–0.97). Simliarly, we did not find any statistically significant association with CVD mortality when lifestyle OBS was treated as quartiles.

The association between dietary OBS and the risk of all-cause and CVD mortality was observed in Supplementary Table 2. When dietary OBS was calculated as a continous variable in Model 3, no significant relationship was found between dietary OBS and all-caused mortality. For CVD mortality, in the fully adjusted Model 3, a higher dietary OBS tended to show an decreased risk of CVD mortality (HR: 0.95, 95%CI: 0.89–0.99). Even after treating dietary OBS as quartiles, there was still a statistically significant association (Model 3: Quartile 4: HR: 0.33, 95%CI: 0.12–0.90).

### Association of OBS with respiratory-related mortality

The association between OBS and respiratory-related mortality risk in participants is presented in Supplementary Table 3. After adjusting for various models in the continuous OBS model, the results were as follows for Model 1, Model 2, and Model 3, the HR were 0.99 (95% CI: 0.94–1.04), 1.01 (95% CI: 0.95–1.06), and 1.07 (95% CI: 0.99–1.13), respectively. Furthermore, no statistically significant associations with respiratory-related mortality were observed when OBS was categorized into quartiles.

### RCS analysis

**Figure 2:**
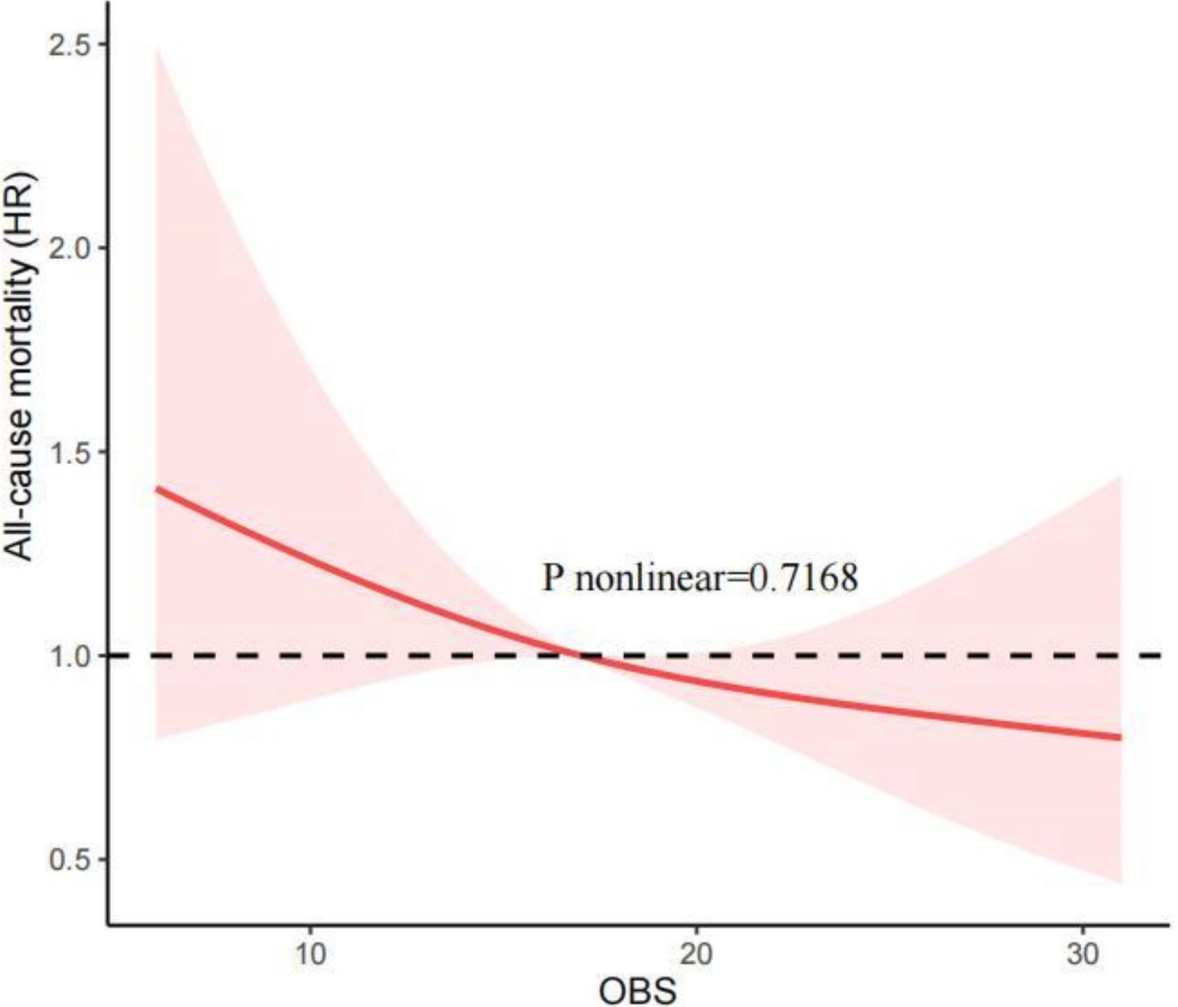
The Restricted cubic spline (RCS) analysis between the OBS and the all-cause mortality.

**Figure 3:**
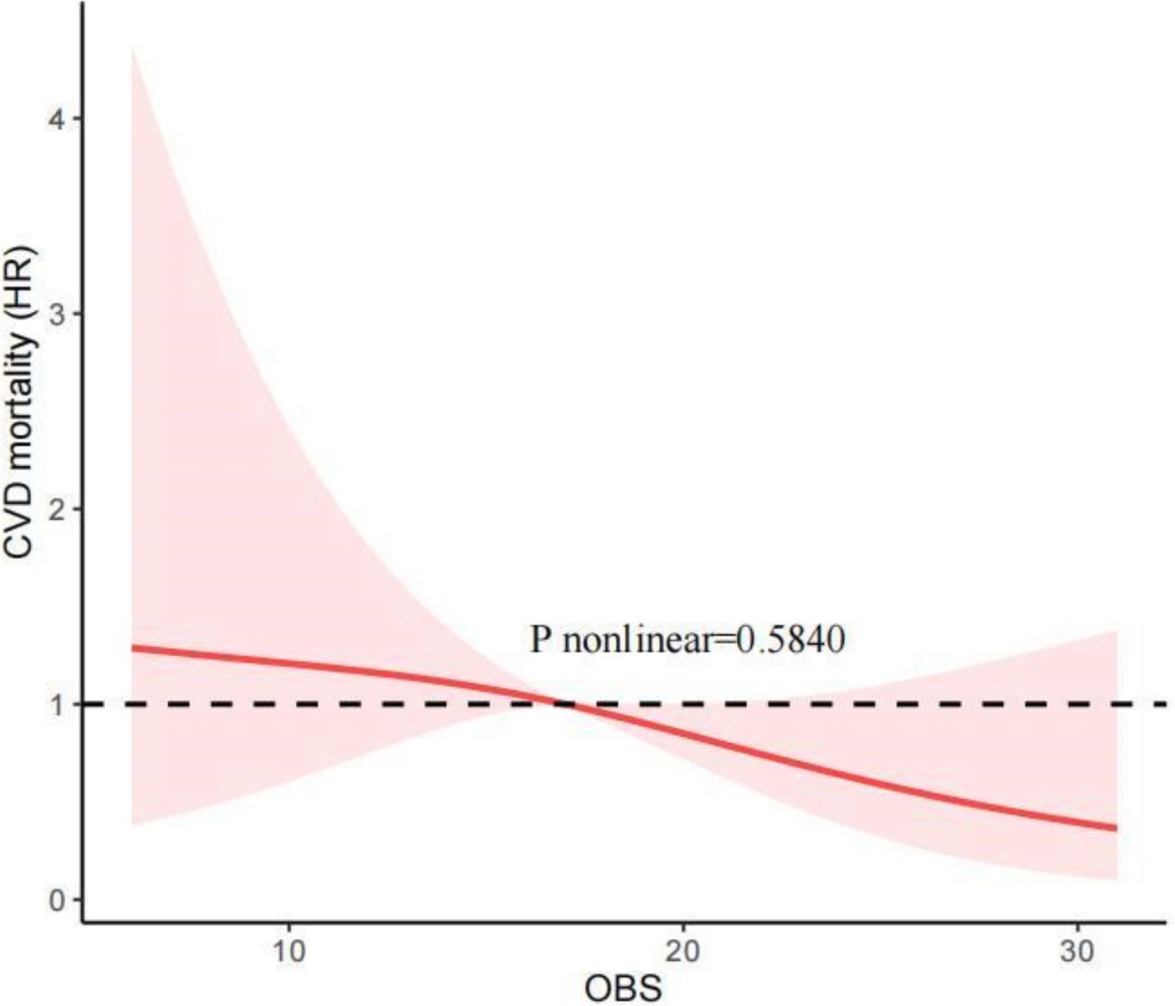
The Restricted cubic spline (RCS) analysis between the OBS and CVD mortality.

Our investigation employed RCS to determine whether the relationship between the OBS and all-cause and CVD mortality was non-linear. Figure 2 shows that, after adjusting for all covariables, the RCS analysis indicated a linear and inverse association between the OBS and all-cause mortality (P nonlinear = 0.7168). Additionally, the RCS analysis also revealed the linear association between the OBS and CVD mortality (*p* nonlinear = 0.5840; Figure 3). These findings confirmed a linear inverse relationship between the OBS and all-cause and CVD mortality in older asthma patients with diabetes.

### Subgroup analysis

**Figure 4:**
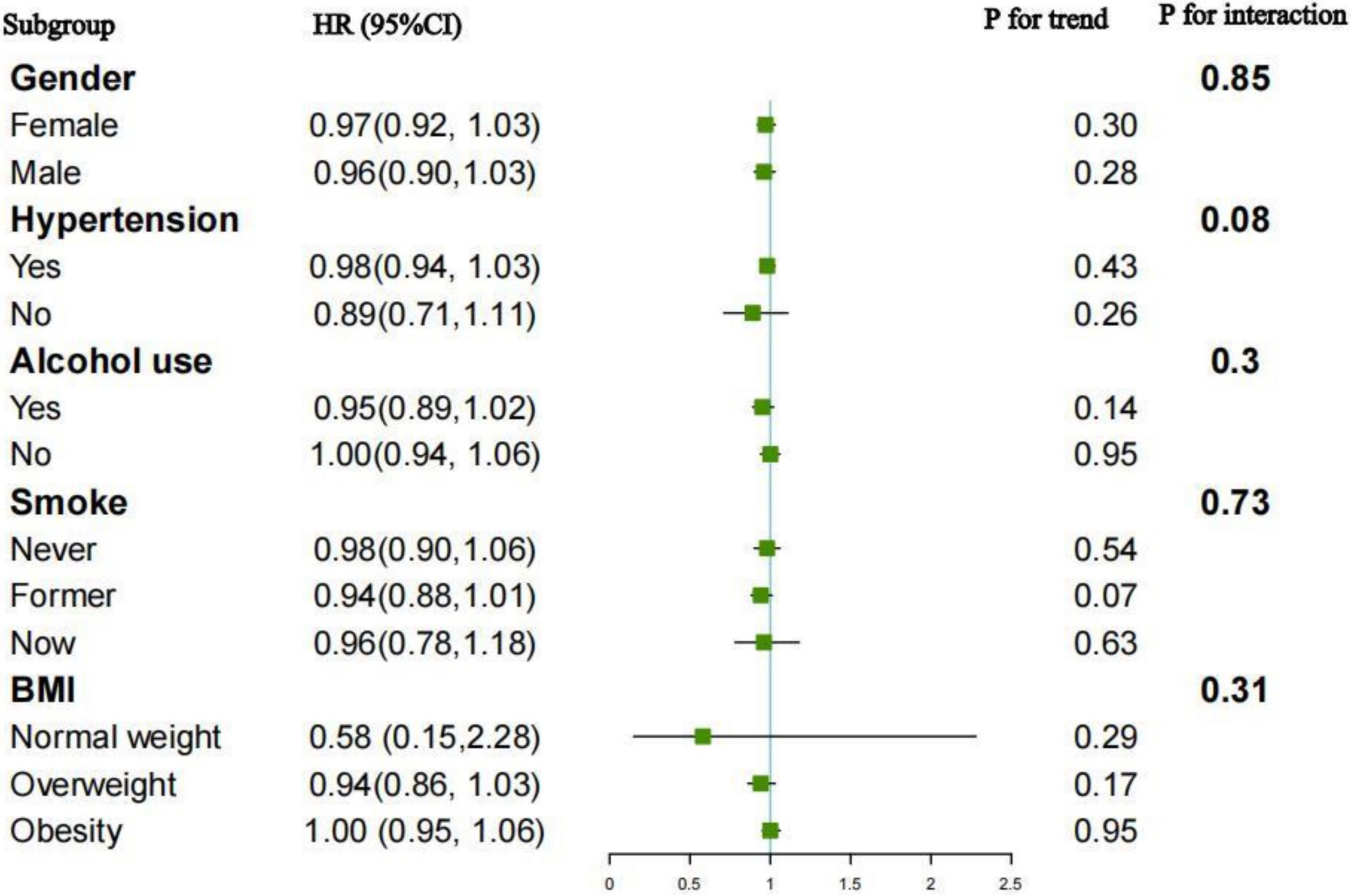
Subgroup analysis for the association between OBS and all-cause mortality.

**Figure 5:**
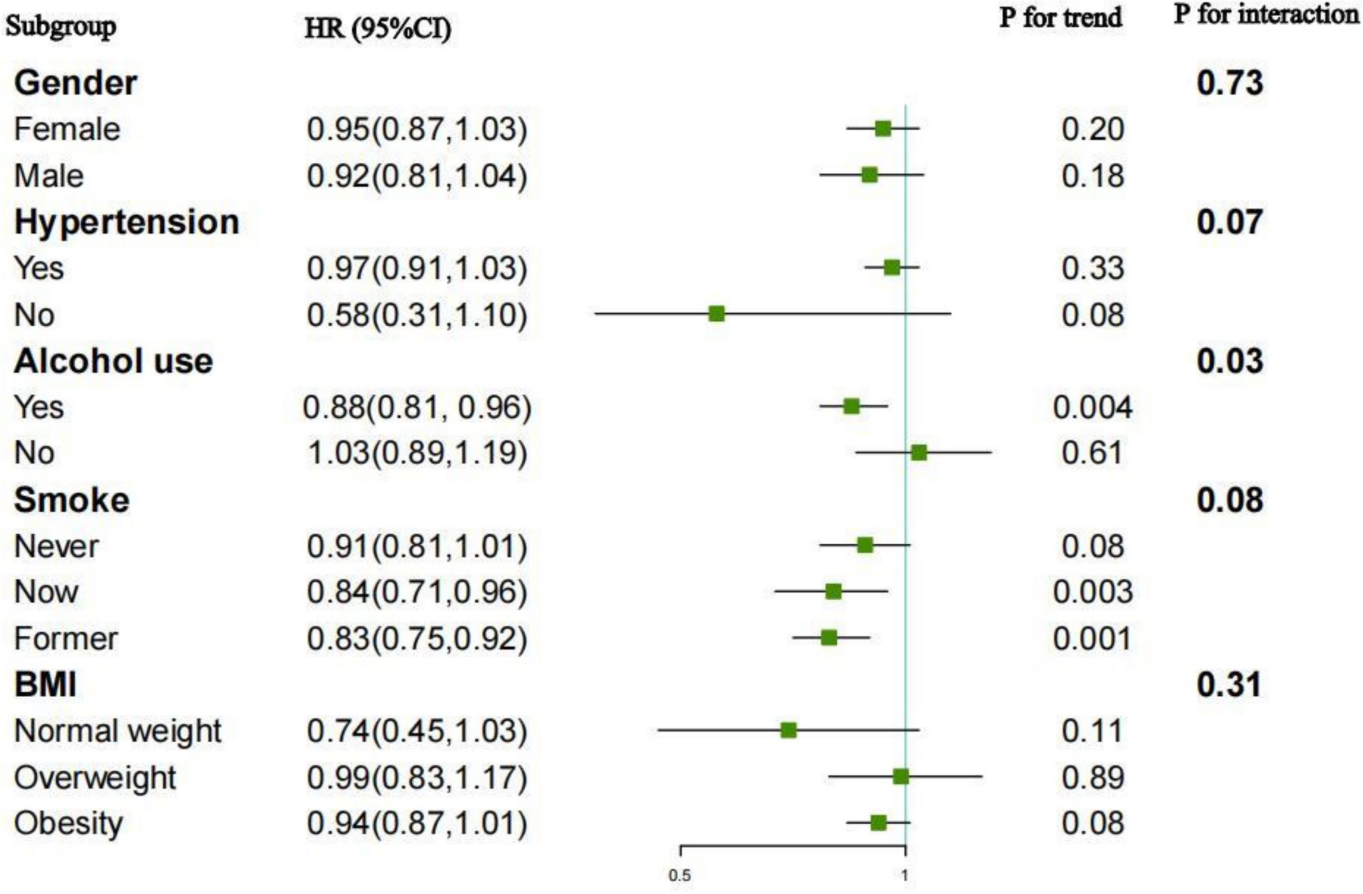
Subgroup analysis for the association between OBS and CVD mortality.

Subgroup analyses and interaction tests were performed to evaluate the relationship between the OBS and all-cause and CVD mortality across different populations. The outcomes, grouped by gender, hypertension, BMI, smoking and alcohol consumption, were displayed in Figures 4, 5. For the association between the OBS and all-cause mortality, we did not observe any statistically significant relationship.

For CVD mortality, a negative association was observed across various subgroups: alcohol users (HR = 0.88, 95%CI: 0.81–0.96), current smokers (HR = 0.84, 95%CI: 0.71–0.96), and former smokers (HR = 0.83, 95%CI: 0.75–0.92). Interaction tests indicated the negative association was significantly influenced by alcohol consumption (*p* for interaction = 0.03). While no significant interactions were observed in other subgroups.

## Discussion

In this study of 611 older asthma patients with diabetes, we identified a negative association between the OBS and all-cause and CVD mortality. However, we did not observe a significant association between OBS and respiratory-related mortality. RCS analysis demonstrated that this inverse relationship was linear. Subgroup analyses and interaction tests indicated the negative association between the OBS and CVD mortality was significantly influenced by alcohol consumption. While the subgroup analyses showed no significant interactions between the OBS and all-cause mortality among the stratification variables. In conclusion, our findings suggest that the OBS is a valuable marker for assessing all-cause and CVD mortality risk in older asthma patients with diabetes and may contribute to the development of effective death prevention strategies. In addition, we also analysed the relationship between lifestyle/dietary OBS and all-cause and CVD mortality. The results showed that lifestyle OBS was negatively associated with all-cause mortality, while dietary OBS was inversely associated with CVD mortality. Last but not least, we did not observe OBS could reduce respiratory-related mortality in older asthma patients with diabetes.

Previous studies have explored the relationship between OBS levels and both all-cause and CVD mortality. Findings from a nationally representative sample indicate that higher OBS may lower the risk of these outcomes, particularly in individuals aged 45 or younger, male, Non-Hispanic White, non-smokers, and those who exercise regularly (24). Another study linked higher OBS to a reduced risk of *H. pylori* infection, with dietary OBS correlating with lower all-cause mortality in *H. pylori*-positive individuals and lifestyle OBS associated with lower mortality in *H. pylori*-negative participants (23). Elevated OBS is also associated with a decreased risk and severity of metabolic syndrome, as well as reduced all-cause mortality in those with the condition (25). Additionally, a positive correlation exists between OBS and reduced prevalence of CVD and mortality in adults with nonalcoholic fatty liver disease (NAFLD), especially among younger and more educated individuals (26). A similar association with reduced all-cause mortality has been observed in older adults (16). In our present study, an independent association was observed between OBS and all-cause and CVD mortality in older asthma patients with diabetes, and RCS analysis revealed a linear negative association between OBS and mortality in older asthma patients with diabetes. Studies have also explored the relationship between OBS and chronic respiratory diseases and lung function. One study found that higher OBS was negatively associated with chronic bronchitis, wheezing, and restrictive spirometry patterns, but positively related to predicted values of FVC and FEV1 (18). Another study in Korean adults showed that OBS significantly improved exertional spirometry (11). However, findings on COPD were mixed; while an increase in OBS was generally linked to a lower prevalence of COPD, excessive OBS showed a non-significant decrease or even increased risk of COPD, indicating an L-shaped association (17).

Diabetes is a significant factor associated with an increased risk of developing asthma. Research indicates that the incidence of asthma is nearly twice as high in individuals with diabetes compared to those without diabetes (27). A large study of Danish twins also found that the risk of asthma was nearly double in patients with Type 2 Diabetes Mellitus (T2DM) compared to non-T2DM patients (28). Additionally, a study of veterans revealed that approximately 5% of diabetics had asthma, compared to 2.9% of healthy controls, regardless of other comorbid conditions (29). Findings from a Taiwanese study similarly showed that individuals with T2D had a 30% higher risk of developing asthma (30). Children with asthma have been found to have higher HbA1c levels compared to healthy controls (31). Asthma can act synergistically with obesity and insulin resistance to increase circulating levels of inflammatory cytokines, which can lead to an increased risk in people with type 2 diabetes (32). In turn, high levels of glucose and insulin in the lungs and low-grade inflammation throughout the body further contribute to collagen deposition and airway remodeling, which leads to decreased lung function in diabetic patients (33–36). Furthermore, asthma management often involves high doses of inhaled or oral glucocorticoids, which can elevate blood glucose levels and potentially accelerate diabetes progression (37). Consequently, asthma is often poorly controlled in diabetic patients, leading to a higher likelihood of disease progression, complications, and hospitalization (38, 39). This chronic systemic inflammation induces a pro-oxidant state, causing an imbalance between pro-oxidant and antioxidant factors, and resulting in excessive production of reactive oxygen species (ROS). OBS, as an indicator of the overall antioxidant/oxidant balance, reflects more comprehensively the overall oxidative stress state of the body, and by evaluating the impact of OBS on all-cause and CVD mortality in older asthmatic patients with comorbid diabetes mellitus, it is possible to make further targeted recommendations for the management of these patients. It is important to note that due to the design of the NHANES study, there is some uncertainty regarding the medications taken by the patients, and therefore more detailed follow-up prospective studies are needed.

In our study, we also observed that compared to never-smokers, current and former smokers benefited more from OBS in reducing CVD mortality. This could be because oxidative stress is a key feature of the pathophysiology of asthma, and components in cigarette smoke, such as nicotine and free radicals, can directly induce the production of ROS in the body (10, 40–43). The accumulation of ROS further exacerbates oxidative stress, leading to cellular and tissue damage, inducing airway hyperreactivity and airway remodeling, which aggravates asthma. Furthermore, in smokers, prolonged elevation of serum CRP and interleukin-6 (IL-6) concentrations is closely associated with reduced lung function (44). Increased oxidative stress due to smoking can also lead to tissue damage through lipid peroxidation (45). Some studies have found that treatments aimed at reducing pro-inflammatory factors and increasing anti-inflammatory factors can reduce the risk of chronic airway inflammation in current and former smokers. However, these treatments did not show a statistically significant protective effect for never-smokers against the risk of chronic airway inflammation (46–48). The OBS includes both oxidative stress and antioxidant defenses. For smokers, interventions that improve antioxidant status (through diet, supplementation, or lifestyle changes) can be more effective at reducing oxidative damage and its negative health consequences. Former smokers still carry a higher oxidative burden than never smokers, as the effects of smoking linger in the body even after quitting. However, over time, former smokers often experience improvements in oxidative balance due to the reduction in exposure to tobacco smoke. Current smokers have the highest levels of oxidative stress, as they are continuously exposed to the harmful substances in tobacco smoke (40, 49). The OBS can be an effective measure for identifying individuals at high risk and motivating interventions that help balance oxidative stress and antioxidant levels. Never smokers generally have a lower baseline level of oxidative stress compared to smokers, so the potential for improvement in oxidative balance is less pronounced. This is why the OBS is less effective in reducing mortality rates in never smokers; they already start from a more balanced oxidative state. In summary, the oxidative balance score is particularly effective in reducing all-cause and CVD mortality among current and former smokers because these individuals experience higher levels of oxidative stress due to smoking. Addressing oxidative imbalance through lifestyle changes and antioxidant support can significantly lower their risk of developing chronic diseases and improve long-term health outcomes. Never smokers, on the other hand, generally start with a more balanced oxidative state, making the OBS less impactful in their case.

This study has several notable strengths. Firstly, we utilized nationally representative data from the NHANES database, which is derived from a national population sample and collected using standardized protocols and stratified, multistage sampling methods, ensuring a representative non-institutional population. Secondly, we took measures to minimize potential biases. Thirdly, our research contributes significantly by examining the association between OBS and all-cause as well as cardiovascular mortality among older U.S. asthmatics with comorbid diabetes. Finally, we conducted various sensitivity and subgroup analyses to further enhance the robustness of our findings.

This study has several limitations. First, many variables in the NHANES were self-reported, which may undermine the credibility of our findings due to the potential inaccuracy of these reports compared to objective records. Second, our analysis is based on observational data, which does not directly address the mechanisms by which the OBS affects mortality in older asthmatics with comorbid diabetes. Further research is needed to explore the biological link between the OBS and mortality to identify potential therapeutic targets. Additionally, using a single 24-h dietary recall to assess pro-oxidants and antioxidants may not fully capture day-to-day dietary variations, though this method helps reduce recall bias. The presence of unquantified variables in our analysis does not exclude the possibility of unrecognized confounding effects. Finally, causal relationships between variables might be weakened, so our findings should be interpreted with caution. Future prospective cohort studies are needed to confirm these results.

## Conclusion

The investigation proved a linear and inverse relationship between the OBS and all-cause and CVD mortality in older asthma patients with diabetes. In summary, our findings suggest that OBS could be a useful predictor of all-cause and CVD mortality risk in older asthma patients with diabetes. Future studies with larger sample sizes are necessary to further validate the regulation mechanisms of OBS in older asthma patients with diabetes.

## Data Availability

The raw data supporting the conclusions of this article will be made available by the corresponding authors under appropriate request.

## Footnotes

^1^ https://www.cdc.gov/nchs/data-linkage/mortality.htm
