# Supplementary material for: Association between oxidative balance score and all-cause, CVD and respiratory-related mortality in the US older adults of asthma patients with diabetes
Source: Front Nutr. 2025 Jan 15;11:1519570. doi: 10.3389/fnut.2024.1519570 (PMC11775759; doi:10.3389/fnut.2024.1519570)
Supplement: Supplementary file 1 [file Supplementary_file_1.docx]

| **All-cause mortality** |  | **HR (95%CI) P value** |  |
| --- | --- | --- | --- |
|  | Model 1 | Model 2 | Model 3 |
| Lifestyle OBS (continuous) | 0.74 (0.66, 0.84) <**0.0001** | 0.70 (0.61, 0.80) <**0.0001** | 0.75 (0.62, 0.91) **0.004** |
| Lifestyle OBS (quartiles) |  |  |  |
| Quartile 1 | Reference | Reference | Reference |
| Quartile 2 | 0.54 (0.37, 0.79) **0.002** | 0.46 (0.30, 0.71) **<0.001** | 0.66 (0.36, 1.18) 0.16 |
| Quartile 3 | 0.37 (0.22, 0.63) **<0.001** | 0.31 (0.19, 0.50)**<0.0001** | 0.39 (0.19, 0.80) **0.01** |
| Quartile 4 | 0.40 (0.24, 0.68) **<0.001** | 0.35 (0.20, 0.59) **<0.001** | 0.47 (0.23, 0.97) **0.04** |
| **CVD mortality** |  | **HR (95%CI) P value** |  |
| Lifestyle OBS (continuous) | 0.75 (0.59, 0.96) **0.02** | 0.71 (0.54, 0.95) **0.02** | 0.88 (0.61, 1.28) 0.51 |
| Lifestyle OBS (quartiles) |  |  |  |
| Quartile 1 | Reference | Reference | Reference |
| Quartile 2 | 0.54 (0.28, 1.07) 0.08 | 0.42 (0.21, 0.85) **0.02** | 0.62 (0.25, 1.55) 0.31 |
| Quartile 3 | 0.52 (0.21, 1.25) 0.14 | 0.51 (0.24, 1.08) 0.08 | 0.69 (0.28, 1.71) 0.42 |
| Quartile 4 | 0.30 (0.08, 1.14) 0.08 | 0.27 (0.06, 1.14) 0.08 | 0.55 (0.08, 3.79) 0.54 |

**Supplemental Table 1 HRs (95%CI) for mortality according to the lifestyle OBS.**

HR: hazard ratio; 95%CI: 95% Confidence Interval

Model 1 was unadjusted; Model 2 adjusted for age, gender, and race; Model 3 adjusted for age, gender, race, education, PIR, BMI, hypertension, total cholesterol, alcohol use, and smoking status
