# Supplementary material for: Association between oxidative balance score and all-cause, CVD and respiratory-related mortality in the US older adults of asthma patients with diabetes
Source: Front Nutr. 2025 Jan 15;11:1519570. doi: 10.3389/fnut.2024.1519570 (PMC11775759; doi:10.3389/fnut.2024.1519570)
Supplement: Supplementary file 2 [file Supplementary_file_2.docx]

| **All-cause mortality** |  | **HR (95%CI) P value** |  |
| --- | --- | --- | --- |
|  | Model 1 | Model 2 | Model 3 |
| Dietary OBS (continuous) | 0.75 (0.59, 0.96) **0.02** | 0.71 (0.54, 0.95) **0.02** | 0.88 (0.61, 1.28) 0.51 |
| Dietary OBS (quartiles) |  |  |  |
| Quartile 1 | Reference | Reference | Reference |
| Quartile 2 | 0.54 (0.28, 1.07) 0.08 | 0.42 (0.21, 0.85) **0.02** | 0.62 (0.25, 1.55) 0.31 |
| Quartile 3 | 0.52 (0.21, 1.25) 0.14 | 0.51 (0.24, 1.08) 0.08 | 0.69 (0.28, 1.71) 0.42 |
| Quartile 4 | 0.30 (0.08, 1.14) 0.08 | 0.27 (0.06, 1.14) 0.08 | 0.55 (0.08, 3.79) 0.54 |
| **CVD mortality** |  | **HR (95%CI) P value** |  |
| Dietary OBS (continuous) | 0.95 (0.91, 0.99) **0.01** | 0.94 (0.89, 0.99) **0.01** | 0.95 (0.89, 0.99) **0.04** |
| Dietary OBS (quartiles) |  |  |  |
| Quartile 1 | Reference | Reference | Reference |
| Quartile 2 | 0.69 (0.29, 1.61) 0.39 | 0.52 (0.25, 1.10) 0.09 | 0.46 (0.19, 1.16) 0.10 |
| Quartile 3 | 0.50 (0.22, 1.14) 0.10 | 0.48 (0.21, 1.13) 0.09 | 0.62 (0.22, 1.77) 0.37 |
| Quartile 4 | 0.38(0.14, 0.99) **0.04** | 0.34 (0.14, 0.87) **0.02** | 0.33 (0.12, 0.90) **0.03** |

**Supplemental Table 2 HRs (95%CI) for mortality according to the dietary OBS.**

HR: hazard ratio; 95%CI: 95% Confidence Interval

Model 1 was unadjusted; Model 2 adjusted for age, gender, and race; Model 3 adjusted for age, gender, race, education, PIR, BMI, hypertension, total cholesterol, alcohol use, and smoking status
