# Supplementary material for: Association between oxidative balance score and all-cause, CVD and respiratory-related mortality in the US older adults of asthma patients with diabetes
Source: Front Nutr. 2025 Jan 15;11:1519570. doi: 10.3389/fnut.2024.1519570 (PMC11775759; doi:10.3389/fnut.2024.1519570)
Supplement: Supplementary file 3 [file Supplementary_file_3.docx]

|  |  | **HR (95%CI) P value** |  |
| --- | --- | --- | --- |
|  | Model 1 | Model 2 | Model 3 |
| **Respiratory-related mortality** |  |  |  |
| OBS (continuous) | 0.99 (0.94, 1.04) 0.71 | 1.01 (0.95, 1.06) 0.80 | 1.07 (0.99, 1.13) 0.05 |
| OBS (quartiles) |  |  |  |
| Quartile 1 | Reference | Reference | Reference |
| Quartile 2 | 0.57 (0.18, 1.78) 0.34 | 0.56 (0.16, 1.93) 0.36 | 0.80 (0.21, 3.02) 0.74 |
| Quartile 3 | 1.58 (0.68, 3.67) 0.28 | 1.27 (0.40, 4.04) 0.68 | 2.73 (0.67, 11.24) 0.16 |
| Quartile 4 | 0.67 (0.19, 2.41) 0.54 | 0.59 (0.19, 1.86) **<0.0001** | 1.17 (0.50, 5.75) 0.39 |
|  |  |  |  |

**Supplemental Table 3 HRs (95%CI) for respiratory-related mortality according to the OBS.**

HR: hazard ratio; 95%CI: 95% Confidence Interval

Model 1 was unadjusted; Model 2 adjusted for age, gender, and race; Model 3 adjusted for age, gender, race, education, PIR, BMI, hypertension, total cholesterol, alcohol use, and smoking status
